# Interceptive Correction of Anterior Crossbite Using Short-Span Wire-Fixed Orthodontic Appliance: A Report of Three Cases

**DOI:** 10.1155/2018/4323945

**Published:** 2018-04-29

**Authors:** S. Nagarajan M. P. Sockalingam, Khairil Aznan Mohamed Khan, Elavarasi Kuppusamy

**Affiliations:** Centre for Family Oral Health, Faculty of Dentistry, The National University of Malaysia (UKM), Bangi, Malaysia

## Abstract

Anterior crossbite is relatively a common presentation in the mixed dentition stage. If left untreated, it can lead to a host of problems and may complicate future orthodontic treatment. One of the major difficulties in performing anterior crossbite correction in young children is treatment compliance. In most cases, poor compliance is due to the unacceptability of the removable appliance used. This article describes three cases of successful correction of anterior crossbite of patients in mixed dentition using short-span wire-fixed orthodontic appliances. This sectional appliance provides an alternative method of correcting anterior crossbite of dental origin and offers many advantages compared to the use of removable appliances.

## 1. Introduction

Anterior crossbite is defined as an abnormal reversed relationship of a tooth or teeth to the opposing teeth in the buccolingual or labiolingual direction, and it is also known as reverse articulation [1]. The prevalence of anterior crossbite ranges from 4.5% to 9.5% based on the respective studied populations [2–5]. In children with malocclusion, it is reported to be around 27% [6].

Many factors may contribute toward the development of anterior crossbite, and the contributory factors can be categorised based on the nature of the crossbite into skeletal, dental, and functional entities [7]. Skeletal anterior crossbite arises due to either genetic or hereditary influence or discrepancy in the size of the maxilla and mandible. The skeletal entity usually involves a segment of maxillary teeth that are proclined at normal angulation but positioned behind the mandibular incisors. In the anterior crossbite of dental origin, one or two teeth are often involved, and the affected tooth/teeth are either upright or retrocline without any significant maxilla-mandible discrepancy. In the functional-type crossbite, a premature contact between the opposing tooth/teeth could result in the deflection of the mandible to the sides or anteriorly, and this leads to the development of pseudoclass-III [8].

Anterior crossbite may give rise to enamel wear mainly close to the incisal edge due to heavy contact between the opposing tooth/teeth [6]. An abnormal bite between the opposing teeth can also affect periodontal health, and this could lead to the gingival recession with thinning of the alveolar bone and mobility of the opposing mandibular tooth/teeth [6, 9, 10]. Functional crossbite due to the premature contact could lead to a possible jaw deviation and temporomandibular pain dysfunction [6, 11].

Many treatment modalities ranging from simple to complex means are available to correct anterior crossbite; some use removable appliances and others use fixed appliances [7, 12–20]. The appropriate method to treat anterior crossbite will depend on the aetiology of the crossbite, the patient's age and compliance, eruption status of the teeth, space availability, and treatment affordability. A simple method such as tongue blade can be used in the early stages of anterior crossbite development as the tooth/teeth are erupting. Appliances such as Catlan's appliance and removable appliances with z-spring(s) or expansion screw or microscrew(s) are often used to correct anterior crossbite related to dental factors in the preadolescent age group. Crossbite of skeletal origin often requires complex methods, such as rapid maxillary expansion and Frankel III appliances. Occasionally, use of extra-oral devices such as a face mask and a chin cup may be necessary to correct the skeletal-based anterior crossbite [7].

This article highlights three cases of successful correction of anterior crossbite using simple short-span wire-fixed orthodontic appliances. The use of this type of appliance provides an alternative treatment modality to correct anterior crossbite with good patient compliance and minimal disruption of oral functions.

## 2. Case Report

### 2.1. Case 1

An 8-year 5-month old boy came with his parents to the Paediatric Dental Clinic of the Dental Faculty at the National University of Malaysia (UKM) with a primary complaint of maligned teeth. Parents noticed that some of their son's upper teeth were behind his lower teeth. The patient has no previous history of dental treatment, and his medical history was noncontributory.

**Figure 1:**
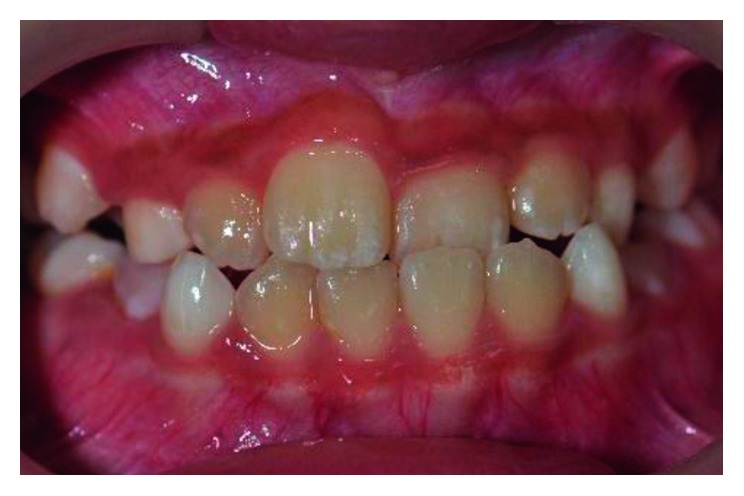
Pretreatment photograph of tooth 12, 21, and 22 in crossbite.

Intraoral examination revealed the patient in mixed dentition stage with the first permanent molars in a Class I relationship. Three of his permanent maxillary teeth, right lateral incisor (tooth 12), left central incisor (tooth 21), and left lateral incisor (tooth 22), were in a crossbite relationship (Figure 1). Slight enamel attrition was noted on the labial surface of tooth 22 close to the incisal edge due to traumatic occlusion. Space analysis using the Moyer's mixed dentition analysis showed the availability of adequate space within the arch for realignment of teeth.

**Figure 2:**
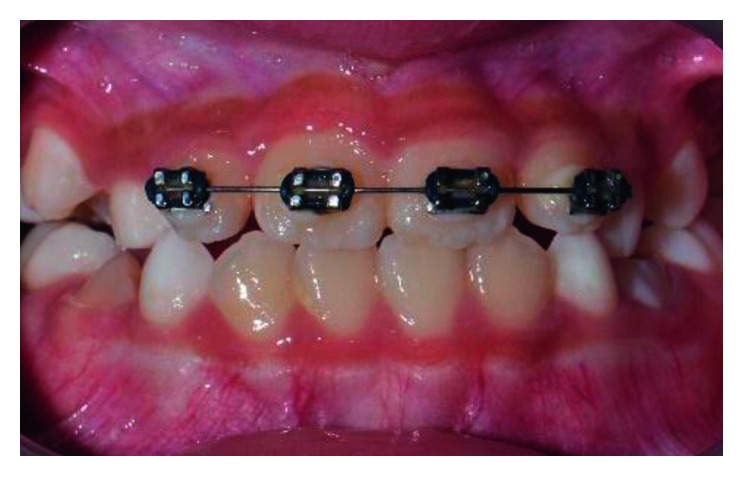
Sectional short-span wire-fixed orthodontic appliance in place during treatment.

After discussing the treatment modalities with parents, we selected a short-span wire-fixed orthodontic treatment with four preadjusted edgewise brackets with a 0.022″ slot. The brackets were bonded on the labial aspects of the four maxillary permanent incisors. A short-span nickel-titanium (Ni-Ti) 0.014″ round archwire is cut equally on both sides of the centreline and placed into the bracket slots (Figure 2). The wire was stabilised in its position using elastic ties. The patient's bite was raised using 2 mm thickness of glass ionomer cement (GIC) placed on the occlusal aspects of both the mandibular first permanent molars (tooth 36 and tooth 46).

**Figure 3:**
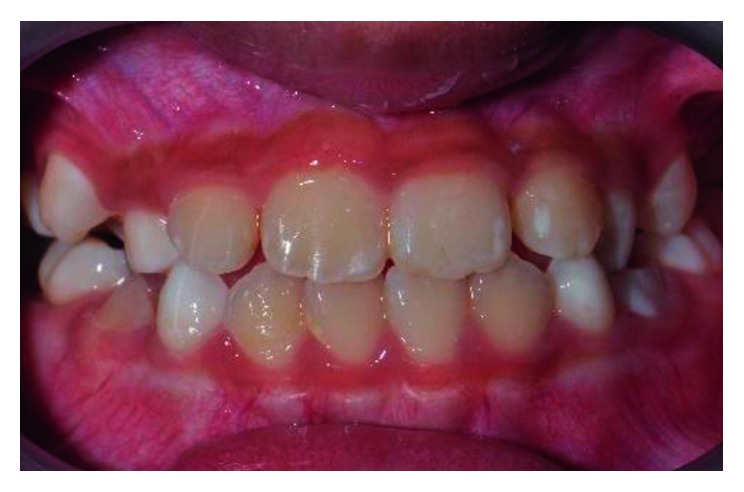
Posttreatment photograph at 3-month review after correction of the anterior crossbite.

Two weeks later, there was some evidence of anterior movement of the maxillary teeth that were in crossbite. Within a month after the initiation of treatment, the anterior crossbite was corrected successfully. The 0.014″ round Ni-Ti archwire was changed to the 0.016″ round Ni-Ti archwire and retained for further two weeks before debonding of the brackets. At 3-month review, the incisor teeth were still in positive overjet (Figure 3).

### 2.2. Case 2

A 7-year 2-month old boy was seen in the Paediatric Clinic at the Faculty of Dentistry, National University of Malaysia (UKM), for routine dental assessment. He had previous dental treatment under general anaesthesia two years ago, and his medical history was noncontributory.

**Figure 4:**
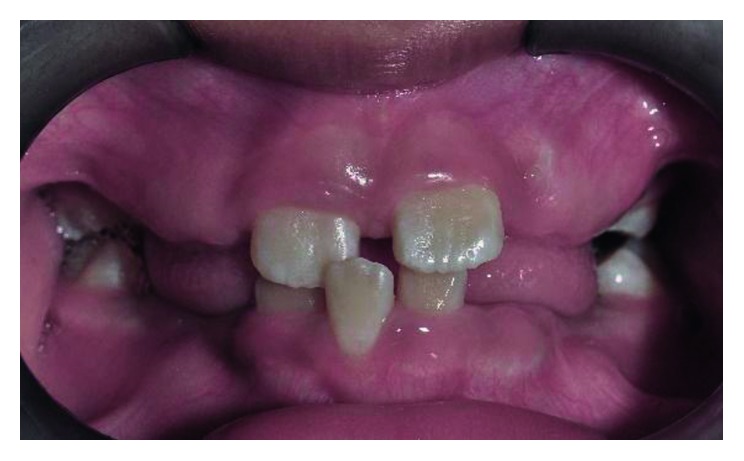
Pretreatment photograph of tooth 11 in crossbite.

Intraoral examination showed all primary teeth of the patient missing due to the previous extraction. Both the permanent maxillary and mandibular first molars on either side have erupted into occlusion. Anteriorly, the permanent maxillary right central incisor (tooth 11) was in a crossbite with the permanent mandibular right central incisor (tooth 41). In occlusion, tooth 11 was trapped between tooth 41 and the permanent mandibular right lateral incisor (tooth 42) (Figure 4). Tooth 41 has Class II tooth mobility and gingival recession on its labial aspect.

**Figure 5:**
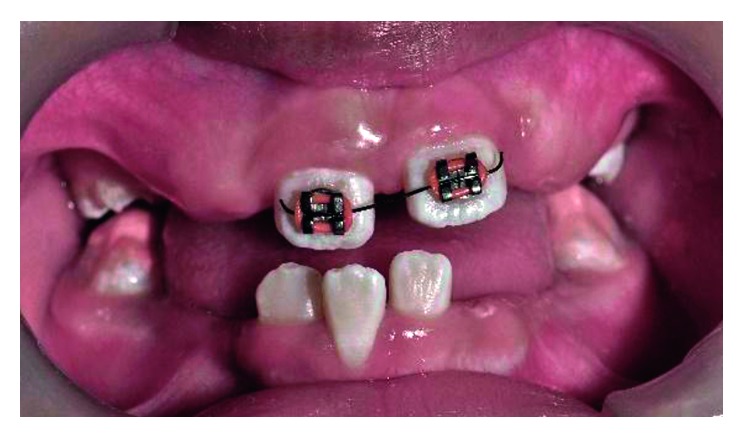
Sectional short-span wire-fixed orthodontic appliance in place during treatment.

After discussion with the parents on the treatment options, we decided on using a short-span wire-fixed appliance with two preadjusted edgewise brackets. The patient's bite was raised with 2 mm thickness of GIC placed on the occlusal aspects of the permanent mandibular first molars. GIC placement allowed opening of the anterior bite and released the lock of trapped tooth 11. Two preadjusted edgewise brackets with a 0.022″ slot were bonded to the labial surface of tooth 11 and the permanent maxillary left central incisor (tooth 21). A short Ni-Ti 0.014″ round archwire was placed into the brackets and held in place with elastic ties (Figure 5).

**Figure 6:**
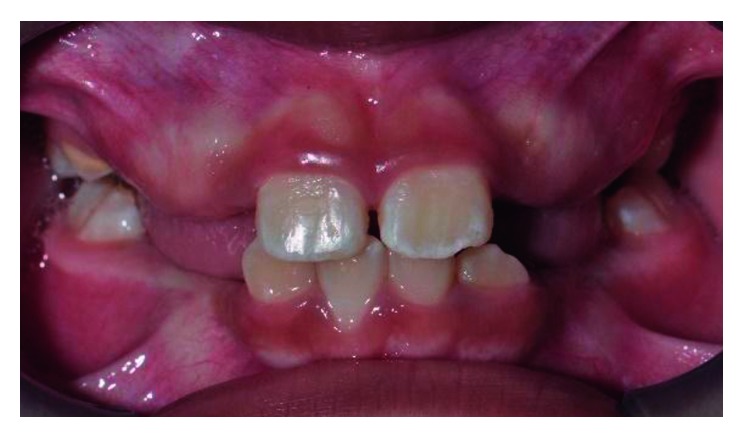
Posttreatment photograph at 6 months after correction of the anterior crossbite.

Two weeks later, the crossbite was corrected. The brackets were debonded, and the GIC on teeth 36 and 46 was removed using an ultrasonic scaler. The occlusion was stable, and the gingival height of tooth 41 showed significant improvement at 6-month review (Figure 6).

### 2.3. Case 3

An 8-year 2-month old boy presented to the Paediatric Dental Clinic of the Dental Faculty at the National University of Malaysia (UKM) with a chief complaint of trapped upper teeth. He had restorative dental treatment to some of his teeth a year ago, and his medical history was noncontributory.

**Figure 7:**
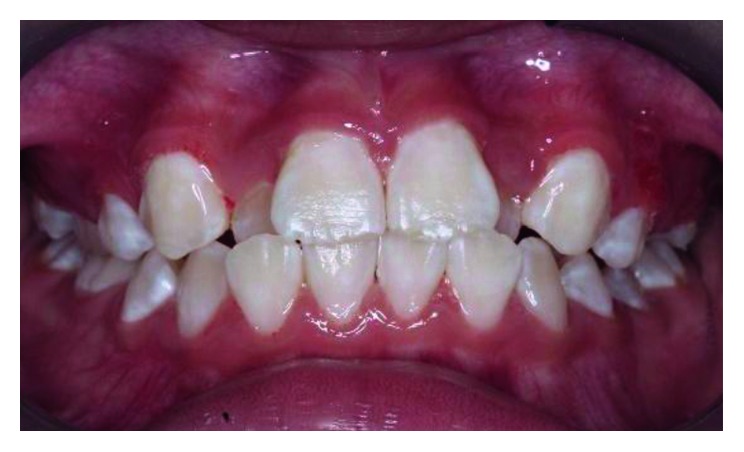
Pretreatment photograph of tooth 12 and 22 in crossbite.

**Figure 8:**
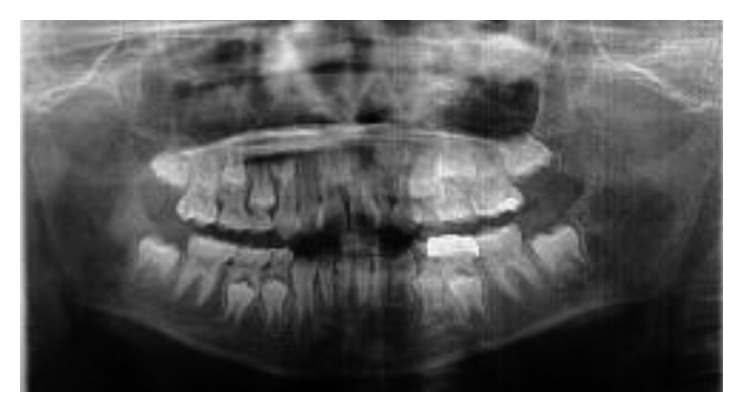
Panoramic radiograph view taken 6 months before treatment showing the position of permanent maxillary canines in relation to their primary predecessors and maxillary permanent lateral incisors.

The intraoral assessment showed that the patient was in his mixed dentition stage and the first permanent molars were in a Class I relationship on either side. The permanent maxillary lateral incisors (teeth 12 and 22) were trapped palatally in an anterior crossbite behind the maxillary deciduous canines (teeth 53 and 63) and the permanent maxillary central incisors (teeth 11 and 21), respectively (Figure 7). He has a Class-I incisor relationship with an overjet and an overbite of 3 mm each. Some evidence of wear facets was noted on the occlusal surfaces of the primary and permanent molars although the patient denied any parafunctional activity. A panoramic radiograph taken a year ago showed the presence of tooth germs of the permanent maxillary canines in a favourable position with no overlapping of the crowns over the roots of teeth 12 and 22 (Figure 8). However, tooth 53 and tooth 63 were relatively big, and limited space was available for teeth 12 and 22 to move anteriorly. Upon consultation with an orthodontist and taking into consideration the patient's molar and incisor relationship, we decided to extract the primary canines to allow distalization of teeth 12 and 22.

**Figure 9:**
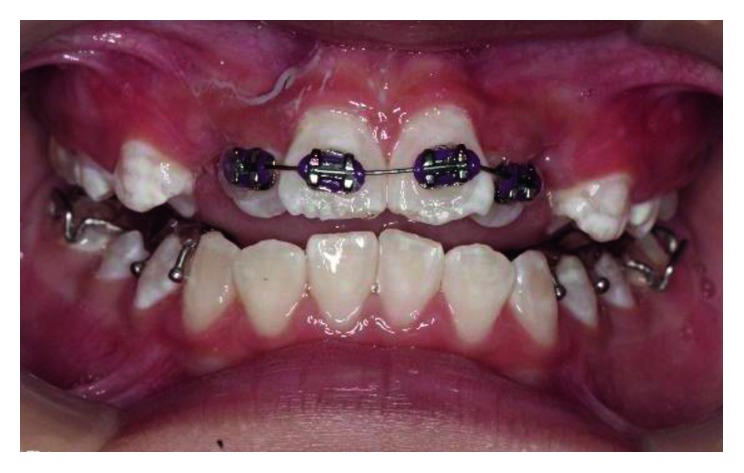
Sectional short-span wire-fixed orthodontic appliance in place during treatment.

**Figure 10:**
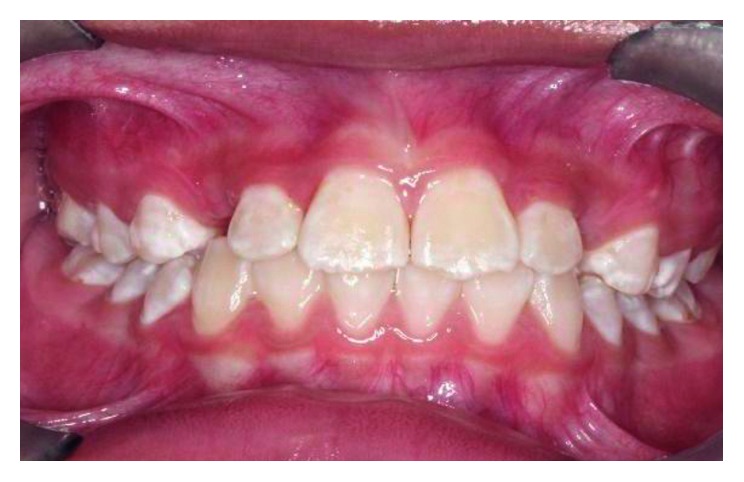
Posttreatment photograph at 6 months after correction of the anterior crossbite.

A month later, after observing slight distalization of teeth 12 and 22, a lower removable bite-raising acrylic appliance was made to open up the anterior bite. Then, four preadjusted edgewise brackets were bonded to the labial surfaces of the maxillary incisors, and a short Ni-Ti 0.014″ round archwire was placed into the brackets and held in place with elastic ties (Figure 9). A month later, anterior movement of teeth 12 and 22 was noted. The existing Ni-Ti wire was changed to the Ni-Ti 0.016″ round archwire, and the patient was reviewed monthly. After three months, we were able to correct the anterior crossbite of teeth 12 and 22. At 6-month review, the corrected teeth were still in positive overjet (Figure 10). The patient is currently under review for the monitoring of the permanent canines eruption.

## 3. Discussion

Anterior crossbite is a common presentation in children during the early mixed dentition stage, and a majority of the cases are of dental origin [21]. Possible causes of dentally related anterior crossbite are the presence of supernumerary tooth/teeth, odontomas, trauma to the primary predecessor, ectopic position of permanent tooth germ, retained primary predecessor, anomalies in tooth shape and size, arch length inadequacy, and upper lip biting habit [7, 13, 14]. These dentally related factors are responsible for deflection of the normal eruption path of the permanent successor tooth/teeth.

Early treatment to correct the anterior crossbite is often advisable to prevent a much more complicated problem and treatment at a later stage. Early treatment allows harmonisation of the occlusion with time, as the permanent teeth are still erupting during this stage of the dentition [15]. However, provision of early treatment has its own sets of problems such as poor patient compliance and refusal of treatment, and the patient may need another phase of orthodontic treatment later. Nevertheless, early treatment can prevent some of the common detrimental effects of anterior crossbite such as enamel wear, gingival striping and attachment loss, tooth mobility, and jaw deviation [16]. Research has shown that patients' oral health quality of life improves with early treatment [22].

Although the use of the intraoral and extraoral appliances can produce the desired tooth or functional jaw movement, patients' compliance very much dictate the treatment success. Common problems encountered with the use of removable appliances include initial speech difficulty due to palatal coverage of the appliance, progressive loosening of the appliance used, and tendency of the patient to flick the loose appliance in and out with the tongue. Besides that, breakage and loss of appliances also happen due to patients' carelessness. Other disadvantages of removable appliances include limited tooth movement range, appliance bulkiness, and poor oral hygiene maintenance. Similarly, patients are also not very much in favour of extraoral devices because of their visibility and social stigma attached to its usage. These adverse effects of both the intraoral and extraoral devices often lead to poor patient compliance and failure of treatment [12, 23].

Use of the fixed orthodontic method to correct anterior crossbite during the preadolescent period has not been widely reported in the literature as compared to other methods as described above. Few cases using a simple fixed orthodontic to correct anterior crossbite and alignment of ectopic teeth have shown good clinical outcome [12, 24, 25]. Many of the problems related to the usage of removable appliances can be overcome with the use of a simple fixed orthodontic appliance. One of the described simple fixed orthodontic appliances is the two-by-four (2 × 4) appliance which allows three-dimensional tooth movement that enables correction of not only the crossbite but also the rotated teeth, teeth with incorrect angulation and inclination, and diastema. Besides that, the 2 × 4 appliance is also suitable for mixed dentition patients with a reduced number of teeth, where the retention of the removable appliance used can be a problem [12, 23, 24, 26].

One of the disadvantages of using the 2 × 4 appliance during the early mixed dentition stage is the placement of bands on the maxillary first permanent molars. Placement of the molar band could be a problem if the permanent molar has not fully erupted or it has a short clinical crown height. Sometimes, placement of the band also can cause discomfort, and some children may refuse further treatment. Furthermore, as the brackets are only bonded to the permanent incisors, there will be a long span of a flexible 0.014″ round Ni-Ti archwire extending from the molar bands to the incisors. The dangling wire can be a problem to the young patients especially during eating and tooth brushing as the wire dangles can easily come out from the molar tube. Another disadvantage of the 2 × 4 appliance is plaque retention around the bands and brackets. However, this could be easily overcome with good oral hygiene care.

The cases presented in this article demonstrated the usage of the sectional short-span wire-fixed orthodontic appliance in correcting cases of anterior crossbite. The appliance is as effective as the 2 × 4 appliance but minus the use of orthodontic bands. The short-span wire-fixed orthodontic appliance method is handy for correction of simple anterior crossbite and especially in cases where the first permanent molars are either unavailable or partially erupted for successful placement of orthodontic bands.

Although this is a simple method for anterior crossbite correction, the clinician should perform a thorough clinical assessment of the patient's facial and dental profiles and make an appropriate diagnosis to determine the cause of the crossbite. The sectional short-span wire-fixed orthodontic appliance is very reliable to correct simple labiolingual discrepancies of the dental origin. However, if the labiolingual difference is vast, use of the 2 × 4 appliance is justifiable because it produces a well-controlled movement of teeth. In anterior crossbite of skeletal origin, sole use of the sectional short-span wire-fixed orthodontic appliance may not produce the desired outcome. Similarly, in functional anterior crossbite, the source of the premature contact needs to be eliminated first before commencing with the correction of the crossbite with either the fixed or removable appliance.

## 4. Conclusion

The highlighted cases showed that it is possible to treat anterior crossbite with the sectional short-span wire-fixed orthodontic appliance, and it offers an alternative treatment option to consider. Early, simple, and tolerable correction of anterior crossbite is beneficial to provide aesthetic and social well-being of the preadolescent children. However, the usage of the sectional short-span wire-fixed orthodontic for treatment of severely rotated teeth, teeth with extreme angulation or inclination, and wide diastema may require further clinical evidence, and consultation with an orthodontist is necessary.
